# Pregnancy Associated Melanoma: Diagnostic and Therapeutic Challenges

**DOI:** 10.3390/medicina62040642

**Published:** 2026-03-27

**Authors:** Vlad-Petre Atanasescu, Ioana-Emanuela Atanasescu, Claudia Mehedintu, Marius Razvan Ristea, Adrian Nicolae Alexandru, Ioana Mihaela Dogaru, Bianca Mihaela Boga, Ana-Maria Oproiu

**Affiliations:** 1 Faculty of General Medicine, “Carol Davila” University of Medicine and Pharmacy, 050474 Bucharest, Romania; vlad-petre.atanasescu@drd.umfcd.ro (V.-P.A.); claudia.mehedintu@umfcd.ro (C.M.); marius-razvan.ristea@drd.umfcd.ro (M.R.R.); adrian.alexandru@umfcd.ro (A.N.A.); ioana.m.dogaru@gmail.com (I.M.D.); bianca-mihaela.boga@drd.umfcd.ro (B.M.B.); anamaria.oproiu@umfcd.ro (A.-M.O.); 2 Emergency University Hospital Bucharest, Department of Plastic and Reconstructive Surgery, Faculty of Medicine (Faculty of General Medicine), “Carol Davila” University of Medicine and Pharmacy, 050098 Bucharest, Romania; 3 Filantropia Clinical Hospital, Department of Obstetrics and Gynecology, Faculty of Medicine (Faculty of General Medicine), “Carol Davila” University of Medicine and Pharmacy, 011132 Bucharest, Romania

**Keywords:** pregnancy-associated melanoma, melanoma in pregnancy, maternal–fetal outcomes, immune modulation, surgical management, immunotherapy, postpartum melanoma

## Abstract

A rare clinical condition associated with numerous diagnostic and treatment challenges, pregnancy-associated melanoma (PAM), is defined as melanoma diagnosed either during pregnancy or within the first year postpartum. The physiological changes in pregnancy (hormonal changes and immune modulation), along with the normal changes in the pregnant woman’s skin (skin color changes, etc.), may all hinder early detection of this disease and create concerns regarding the advancement of melanoma and the well-being of both the mother and her fetus. The purpose of this review article was to summarize the current literature on the incidence, biology, diagnostic methods and treatments of PAM, with an emphasis on comparison between the two forms of melanoma. More recent research indicates that pregnancy itself is not typically associated with decreased melanoma-specific survival rates. However, when worse results are reported, it appears that this may be more due to delays in initial diagnoses (diagnosis of cancer after delivery) or detection of cancer postpartum, as well as the increased number of stages of melanoma at which women were diagnosed at the time of their first evaluation compared to non-pregnant controls, rather than being a result of enhanced biologic aggressiveness in melanoma driven by pregnancy itself. The preclinical and translational models have suggested that pregnancy may influence melanoma biology through the mechanisms of hormonal signaling, immune system modulation and vascular remodeling; however, these mechanisms remain hypothesis-generating, and current clinical evidence does not indicate that changes in hormone levels during pregnancy negatively affect melanoma survival. Surgical excision is the mainstay of treatment and can be performed safely during pregnancy. In select patients, a sentinel lymph node biopsy may also be performed. Due to the risk of fetal harm, systemic therapy (targeted agents and/or immune checkpoint inhibitors) cannot be used for the treatment of PAM during pregnancy. Post-pregnancy treatment of PAM will follow standard melanoma treatment guidelines; however, the treatment options will need to take into consideration whether or not the patient is breastfeeding and if she desires to become pregnant again in the future. In summary, PAM will require a multidisciplinary, individualized approach to maximize oncologic outcomes while protecting the health of both the mother and her fetus. Awareness of this disease and timely diagnosis are critical to maximizing the prognosis.

## 1. Introduction

The relationship between pregnancy-associated melanoma and poor outcomes remains controversial. Some research indicates that there may be a worse prognosis and higher mortality rates associated with melanomas diagnosed during pregnancy, while other studies do not demonstrate a significant difference in outcomes when comparing melanomas diagnosed during pregnancy versus those diagnosed in non-pregnant women [1,2]. Therefore, it is reasonable to assume that there is some level of interaction between the physiologic changes in pregnancy and the potential for melanoma to progress. Therefore, a thorough evaluation of all current research would help determine the actual nature of the relationship between pregnancy and melanoma [3,4].

The association between pregnancy and the prognosis of melanoma has been a topic of debate. The previous literature indicated that alterations in hormone levels, immune status, and blood vessels during pregnancy can affect how melanomas behave (i.e., their biological characteristics). However, there is substantial heterogeneity in the data that exists regarding this issue. Therefore, interpretation of the data must be cautious.

In vitro studies, studies examining the expression of receptors in tumors, and studies using animal models, have identified biologically feasible mechanisms by which pregnancy can alter melanoma biology. These studies demonstrate the potential for pregnancy to affect the biology of melanoma; however, they do not demonstrate a significant adverse effect on the prognosis of women who are pregnant with melanoma [5,6,7].

Recent clinical studies and current reviews of the literature suggest that there is no consistent association between pregnancy and poor outcomes related to melanoma when the stage at time of diagnosis and other prognostic variables are taken into consideration. Therefore, the central question is not whether melanoma is definitively “hormone responsive” in the clinical setting, but whether measurable prognostic consequences exist because of pregnancy-induced physiological changes in humans. There is no evidence to support an adverse prognostic effect of pregnancy alone; however, it is possible that delayed diagnosis or postpartum presentation may contribute to what appear to be poorer outcomes in some series [8,9]. Pregnant patients with melanoma should therefore be treated in accordance with established oncologic standards with modifications as necessary to ensure fetal safety, take into account gestational age, and accommodate the preferences of the mother [10].

A multidisciplinary team of experts is recommended to ensure that treatment decisions are made with appropriate expertise and knowledge of both the disease process and the pregnancy. It is also important to consider delivering the patient at a hospital that is equipped to handle high-risk pregnancies to protect the health and well-being of the developing fetus [11].

## 2. Purpose, Methods, and Organization of This Review

The objective of this review is to undertake a full and accurate summary of the most current information on pregnancy-associated melanoma, including how often it occurs, what it looks like under microscopic examination, the issues encountered when attempting to make an accurate diagnosis of melanoma during pregnancy, how melanoma is treated in pregnant patients and the potential impacts on both the mother and fetus. In addition to these objectives, this review will critically evaluate the methodologies employed and results from the most recently published studies to assess areas of agreement and areas of disagreement. The overall objective of this approach is to improve the way clinicians treat patients who have melanoma and also assist researchers with developing new avenues of study in this difficult and high-risk field that represents an overlap of obstetric/maternity care and oncology. Additionally, this review also examines whether pregnancy itself represents an independent prognostic factor in melanoma. A thorough analysis of existing data will be required to answer this question. Further, this review will analyze the molecular/cellular changes that occur as a result of pregnancy and if the alterations in hormone levels and the immunological status of the patient influence the behavior/growth of the tumor during pregnancy.

A structured literature search using the electronic databases PubMed, Scopus, and Web of Science was used to identify research studies on pregnancy-associated melanoma. A structured search strategy was developed, including English language publications from 2000 to 2024, with a combination of the following key words: pregnancy-associated melanoma; melanoma during pregnancy; melanoma management in pregnancy; melanoma diagnosis in pregnancy; and maternal melanoma outcomes. Articles were included if they described melanoma that occurred during pregnancy or within one year after delivery, and investigated the epidemiologic, pathophysiologic, diagnostic, or therapeutic aspects of melanoma associated with pregnancy. Clinical studies, systematic reviews, and meta-analyses were also considered eligible types of studies. Articles that did not meet these inclusion criteria were excluded from this review, based on the following exclusion criteria: non-English publications; conference abstracts without full text availability; and studies that did not include information about melanoma occurring during pregnancy. The titles and abstracts of the potential articles that met the inclusion criteria were first reviewed to determine their relevance, and then a full-text review of all remaining articles was performed. Additionally, references listed in selected articles were manually searched for additional relevant articles.

As a narrative-based review, this review has the same limitations as all other non-systematic reviews of the literature (i.e., possible selective bias and differences among studies).

## 3. Epidemiology and Risk Factors

In recent years, there has been an increase in the number of women diagnosed with melanoma during pregnancy. According to epidemiologists, CMM is currently the most common type of solid tumor found in pregnant women. Approximately one-third of all female melanoma cases occur during this stage of life, which means that melanoma disproportionately occurs in women of reproductive age [10].

There has been an upward trend in melanoma incidence among women of childbearing age. This trend indicates the increasing importance of pregnancy-associated melanoma (PAM) clinically [3,5].

Melanoma is formed by melanocytes; these are pigment-forming cells that can be found in the basal layer of the epidermis. Most melanomas develop in the skin, but they can be found in mucous membranes and parts of the urogenital tract and GI tract as well, which account for about 5% of non-cutaneous melanoma cases [12].

The SEER Program reports that the five-year survival rate for all stages of cutaneous melanoma is approximately 94%, while the five-year survival rate for metastatic cutaneous melanoma is approximately 39.4% for cases diagnosed between 2016 and 2018 [13].

For patients with metastatic melanoma who were treated with immune checkpoint inhibitors between 2007 and 2018, five-year survival rates varied by the type of melanoma, with a 46% five-year survival rate for cutaneous, a 34% for acral, a 21% for uveal, and a 22% for mucosal melanoma [14].

It appears that the incidence of melanoma diagnosed in pregnancy does not exceed what would be predicted by the estimated prevalence of melanoma in the general population; therefore, it seems that pregnancy alone does not cause melanoma to form [15].

Worldwide, the incidence of cutaneous melanoma has increased faster than almost any other cancer, with an alarming shift towards younger demographics. The median age at time of diagnosis is roughly 57 years, which is significantly younger than that seen in other commonly diagnosed cancers such as prostate, lung, and colorectal cancer [5,16]. While the melanoma incidence is higher in young adult women (aged 20–24), the disease still remains much more prevalent in older adult males (>65 years). Furthermore, melanoma incidence has consistently increased over the last four decades, with over one-third of melanoma cases in women being diagnosed during their reproductive years [10,17].

Despite controversy regarding the long-term outcome implications of PAM, some studies indicate that PAM may be associated with adverse outcomes compared to melanoma diagnosed in non-pregnant individuals. Age-standardized melanoma incidence rates range from 3.5 to 34.9 cases/100,000 females in European countries, and melanoma represents approximately 31% of all solid cancers diagnosed in pregnancy, making it the most common solid cancer diagnosed during pregnancy [18].

However, substantial epidemiologic evidence supports the notion that pregnancy is not independently related to the risk of developing melanoma nor the aggressiveness of the disease. A meta-analysis of ten case–control studies that included >5000 women showed no association between pregnancy and melanoma incidence or progression [19].

Further, multiple large studies have demonstrated no difference in melanoma-related mortality in pregnant women when compared to those who are not pregnant. Women who had a history of at least one previous pregnancy had similar 10-year DFS and OS rates when compared to nulliparous women (94% vs. 83%); however, this was inversely correlated with increasing parity and younger age at first pregnancy, which suggests a protective effect of prior pregnancies [20].

## 4. Pathophysiology

There is no definitive evidence that pregnancy is an independent risk factor for melanoma; however, various pathophysiological factors have been examined for their roles in melanoma development during pregnancy. These factors include: fetal immunological tolerance, the effects of skin changes on cancer diagnosis and delayed detection, the influence of sex hormone levels, enhanced angiogenesis and lymphangiogenesis, which may affect early metastasis to sentinel lymph nodes [11].

### 4.1. Immunologic Changes During Pregnancy

Pregnancy is not an overall suppression of the immune system but instead a dynamic condition of immune regulation, as the mother’s immune system modulates to accommodate the semi-allogenic fetus that expresses paternal antigens. In order to accept the fetus (which can be thought of as an allograft), the maternal immune system will undergo trimester-specific coordinated immunological changes, including preserved or increased innate immunity, expansion of adaptive regulatory mechanisms and suppression of cytotoxic T-cells from responding to certain antigens. The changes that occur are part of the complex metabolic immune re-programming of the immune response and are different in each trimester of pregnancy [21,22,23]. This adaptive environment, in which the immune system can be modulated by both the mother and fetus, is a key component of fetal tolerance. However, because it has been demonstrated that the predominant pathways of regulation are active and that certain cytotoxic T cell responses are attenuated, it has been hypothesized that pregnancy creates conditions under which tumors may potentially have an advantage when it comes to avoiding immune detection. That being said, there is no clinical evidence that demonstrates these physiological immune alterations actually promote progression of melanoma [24,25].

The placental production of indoleamine 2,3-dioxygenase, an enzyme recognized for inhibiting T-lymphocyte proliferation, is postulated to play a role in this immunological tolerance, possibly promoting melanoma progression [26].

The expansion of CD41 CD251 regulatory T cells (Tregs) is essential for fetal survival [5]. Tregs facilitate tolerance throughout both gestation and cancer. These cells are present in cancer and may be associated with diminished antitumor immunity, suppression of effector T lymphocyte proliferation, and increased tumor vascularity. Some researchers say that the immune system changes that happen during pregnancy and the immune system changes that happen when cancer cells grow and spread are similar. However, there is not much evidence that pregnancy causes specific changes in the immune system that lead to malignant melanoma. Scientists are still looking into the specific ways that pregnancy-related immune changes might affect melanoma biology, focusing on how cells and molecules interact to either help tumors escape the immune system or make them more likely to spread [5,24]. The specific mechanisms through which pregnancy-induced immune alterations might influence melanoma biology remain an active area of investigation, focusing on cellular and molecular interactions that could either promote tumor immune escape or enhance metastatic potential. Additional investigation into the function of indolamine 2,3-dioxygenase inhibitors, presently being developed for melanoma therapy, may clarify their capacity to alleviate the immunosuppressive milieu typical of pregnancy, consequently influencing melanoma advancement [10].

Studies show that there is inflammation in the lining of the uterus during embryo implantation, but once the trophoblast forms, it responds by becoming less inflamed. This is meant to help the fetus’s immune system accept it and keep the pregnancy going. Cancer cells can change their appearance to seem like trophoblasts, so that the immune system cannot find them. Moreover, the establishment of tolerance mechanisms to fetal neoantigens, especially those governed by regulatory T lymphocytes, may play a crucial role in facilitating cancer cells to implement tactics that evade immune responses. This immune tolerance generates conditions that allow tumors to develop and advance more quickly because the body does not actively fight them [27,28,29]. This parallel indicates that the maternal immune system’s modifications to accommodate the fetus may unintentionally promote immune evasion by melanoma cells, potentially influencing disease development [5,27].

### 4.2. The Impact of Skin Changes on the Course of Cancer Diagnosis and Delayed Detection

Physiological skin changes during pregnancy can affect both skin color (pigmentation) and blood vessel changes, making it even more difficult to identify abnormal skin lesions, like melanoma, at an earlier stage [30]. Pregnancy can be related to an increase in melanocytic activity, which could possibly be due to an increase in melanocyte-stimulating hormone (MSH). The MSH will bind to the melanocortin-1 receptor (MC1R), a type of G protein-coupled receptor that is found on melanocytes and melanoma cells. Once the MSH binds to MC1R, it will activate the cAMP/PKA signaling pathway. The cAMP/PKA signaling pathway will stimulate the expression of microphthalmia-associated transcription factor (MITF), which regulates the proliferation, differentiation, and survival of melanocytes. Therefore, through this pathway, MSH signaling could affect melanocytic proliferation and survival [31]. Some of the most common hormonal changes during pregnancy cause hyperpigmentation of the skin, specifically melasma and linea nigra. Melasma and linea nigra can cover up small skin changes that could indicate a malignant process [10,17]. Further, pregnant women experience increases in their skin vasculature and erythema (redness). The redness experienced by many pregnant women can interfere with the ability of clinicians to perform a dermatoscopic examination to determine if a lesion is a benign vascular condition or an amelanotic melanoma [10]. The physiological changes in the skin of a woman experiencing pregnancy include striae distensae. Non-ablative fractional laser technology has recently been identified as a safe and effective option for treating striae distensae in young patients [32]. Additionally, the physiological skin changes that occur during pregnancy may decrease clinicians’ clinical suspicion for evaluating previously existing pigmented nevi, thereby decreasing the chances for early diagnosis of melanoma. Early detection of melanoma is significant because size is one of the indicators of how severe the prognosis will be, and larger tumors are typically diagnosed later than smaller ones [26,33,34,35]. Therefore, dermatologic surveillance should continue, and clinicians and patients should increase their awareness to diagnose and treat suspicious pigmented lesions early in the course of pregnancy [35,36].

### 4.3. The Influence of Sex Hormone Levels

The idea that the physiological hormonal changes occurring in women who are pregnant will impact the biological characteristics of melanoma tumors has been researched for many years. Early theoretical ideas suggested that because the concentrations of sex hormones increase in women when they become pregnant, the sex hormones would have an impact upon the biological behaviors of melanoma tumors by interacting through sex hormone-dependent cellular signaling pathways. Melanoma is not recognized as a classic hormone-dependent cancer type, and therefore, the potential utility of these signaling mechanisms in clinical settings remains unclear [5,37]. The mechanism through which estrogen exerts its effects involves binding to estrogen receptors (ERα and ERβ). Studies indicate that the expression of these receptors varies among melanoma cells and is somewhat heterogeneous. Experiments have shown that the activation of these receptors can regulate multiple intracellular signaling cascades that include the PI3K/AKT and MAPK/ERK cascades, which have been implicated in regulating melanoma cell proliferation, survival and apoptosis. Additionally, estrogen signaling has been found in experimental models to stimulate the expression of VEGF, a protein that promotes angiogenesis and thus may contribute to tumor progression [31].

Estrogen signaling has also been reported to modulate the activity of microphthalmia-associated transcription factor (MITF) that is essential to melanocyte differentiation and melanoma cell survival. Loss or dysregulation of MITF activity has been associated with an increase in the ability of melanoma cells to exhibit plasticity and invade other tissues [31].

Expression of the progesterone receptor has been detected in some melanoma tissue samples. However, the significance of the signaling through the progesterone receptor in melanoma is unknown. While there is some experimental evidence that progesterone influences apoptosis and immune responses in the tumor microenvironment, the existence of sufficient evidence to support a conclusion that it directly affects melanoma aggressiveness is currently lacking [38].

Physiological increases in melanocytic activity and hyperpigmentation have been observed in women who are pregnant, which includes common conditions, such as melasma and linea nigra, and less common, but historically noted alterations in benign melanocytic nevi, such as changes in the nevi’s vascularity or perceived growth. Historically, these changes have raised concerns regarding whether these changes reflect an increased risk of malignant transformation. Although these changes are generally accepted as being physiological responses to the vascular and hormonal changes occurring in women during pregnancy, and not indicative of malignant progression, they do represent important issues related to assessing melanoma prognosis [39].

More importantly, while several biologically plausible mechanisms for how sex hormone signaling can influence melanoma biology have been developed based on experimental studies, none of the clinical studies have demonstrated that exposure to the endogenously produced hormonal changes that occur in women during pregnancy independently affects the prognosis of melanoma. Therefore, these studies suggest that hormonal effects may influence melanoma-related signaling pathways in certain experimental conditions, but the clinical implications of these hormonal effects in relation to pregnancy-associated melanoma are unclear [5,26].

### 4.4. Increased Angiogenesis and Lymphangiogenesis

Pregnancy is associated with a pro-angiogenic and pro-lymphangiogenic state characterized by increased vascular endothelial growth factor (VEGF) expression and enhanced lymphatic remodeling [23]. These physiological vascular adaptations, necessary to support placental and fetal development, have been hypothesized to facilitate melanoma dissemination by increasing lymphatic density and potentially promoting metastatic spread [26].

Experimental animal models suggest that pregnancy-associated increases in lymphangiogenesis may promote enhanced metastatic dissemination of melanoma. However, the translation of these findings to clinical outcomes in humans remains uncertain [17,34].

A better understanding of the exact angiogenic and lymphangiogenic pathways activated during pregnancy and their interactions with melanoma cells will provide insight into the mechanisms responsible for metastatic dissemination. Potential mechanisms of interest for melanoma include the angiogenic pathway (VEGF-A and VEGFR-2) and the lymphangiogenic pathway (VEGF-C/VEGF-D and VEGFR-3), along with potentially other pathways related to vascular remodeling during pregnancy, including the angiopoietin/Tie system and the placental growth factor (PlGF)/VEGFR-1 system. While the pathways of interest are both physiologically and pathophysiologically applicable to vascular or lymphatic remodeling, the independent contribution of each pathway to metastasis in pregnant women with melanoma has not been determined [40,41,42,43].

Furthermore, the levels of human chorionic gonadotropin and estrogen increase in the first and second parts of pregnancy. Estrogen levels rise during pregnancy, and estrogen receptor signaling has been implicated in melanoma biology, including modulation of proliferative and angiogenic pathways. Although these mechanisms suggest a potential influence of pregnancy-related hormonal changes on tumor behavior, clinical evidence demonstrating a direct effect on melanoma progression remains limited [38]. Pregnancy is associated with increased lymphangiogenesis, a physiologic process that has been hypothesized to influence tumor dissemination. Experimental and translational studies have suggested that enhanced lymphatic remodeling could theoretically facilitate sentinel lymph node involvement in melanoma. However, current clinical evidence does not demonstrate that pregnancy-associated lymphangiogenesis independently increases the risk of lymphatic metastasis, and this proposed mechanism, therefore, remains speculative [17,26].

A comparative study evaluating lymphangiogenesis and angiogenesis in melanoma tumors from pregnant and non-pregnant women reported a significantly higher number of lymphatic vessels within tumors of pregnant patients. However, although angiogenic factors present within the tumor microenvironment were found to influence melanoma progression, the presence of pregnancy itself did not appear to modify this process. These findings suggest that pregnancy-related lymphatic remodeling may represent a biologic difference in the tumor microenvironment, but current evidence does not demonstrate that it independently increases the risk of metastasis or worsens melanoma prognosis [35]. Therefore, angiogenic and lymphangiogenic changes should be interpreted as potential biologic modifiers rather than proven drivers of adverse prognosis. Further studies correlating these pathways with stage, nodal status, recurrence, and survival are needed before clinical conclusions can be drawn.

## 5. Diagnostic Criteria

Skin pigmentation is the primary sign of pregnancy. Skin pigmentation occurs when the body has high levels of estrogen, progesterone, and melanocorticotropin hormone (MSH). High levels of estrogen stimulate melanocytes to produce melanin. Progesterone supports the production of melanin, thus causing skin pigmentation to occur. Some women will see the effects of skin pigmentation as early as the first trimester of their pregnancy. Specifically, the area around the genitalia and areola will darken. Women will also experience darker freckles, moles or scarring, and in addition, some women may develop chloasma. Chloasma is a condition where women develop large patches of irregularly shaped and defined dark pigmentation on their faces, primarily on the cheeks. In addition, approximately fifty percent of women develop stretch marks in the last two months of their pregnancy. Stretch marks appear as red or blue, indented lines on the abdominal area, breasts and thighs [37].

Due to the many normal changes that occur to a pregnant woman’s skin, doctors may be distracted from diagnosing other problems, which is why doctors need to perform a complete examination and take into account the changes in the patient’s skin. Pregnancy should make doctors concerned about any pigmentary alteration that differs from previous clinical or dermatoscopic findings. Due to the prevalence of hyperpigmentation, the increased number of visits to healthcare providers during pregnancy, and the increased risk of developing thicker melanomas during pregnancy, women should seek immediate attention if they notice any unusual changes in their skin [37].

Studies indicate that the incidence of melanoma is greater in primigravid women than in multiparous women and in women who are diagnosed within twelve months of delivery [35].

When any abnormal skin changes are noticed, the diagnostic process begins immediately. The doctor, such as a family doctor, dermatologist, or gynecologist, uses the ABCDE criteria (asymmetry, borders, color, diameter, and changes over time) to identify the problem. They can also refer the patient for a biopsy of the affected area [44].

There are four general dermatoscopic criteria for identifying nevi. Each criterion is based on specific variables, including: 1. the color of the lesion; 2. the shape of the lesion; 3. the type of pigmentation present in the lesion (such as multifocal, central, eccentric or homogenous); and 4. the location of the lesion, especially in areas of the body that are highly sensitive (such as the face, genitals, nails, and mucous membranes). Dermatologic patterns of pigmentation of individual nevi can vary depending on several patient-related factors, including the patient’s age, skin type, history of melanoma, exposure to ultraviolet radiation, pregnancy status, and developmental stages [45].

Most nevi grow larger in response to the stretched skin and increased blood flow associated with pregnancy [4]. This was shown using dermoscopy and by observing changes in the pigment network or blood vessels [4]. Although nevi removed surgically from pregnant women may show slight degrees of histologic atypia or mitotic activity, this should not be dismissed or considered normal [4]. Therefore, knowing the patient’s medical history, especially if the patient is pregnant and/or the time since the patient gave birth (three to six months), is important when evaluating the patient’s skin to determine the cause of a skin alteration. Even though a clinical–pathological correlation and histologic evaluation are performed, if the clinician still has questions about the benignity or malignancy of the melanocytic lesion, performing molecular studies on the lesion may provide additional evidence of the malignant potential of the lesion. Additionally, any new, changing, or darkening nevi in pregnant women should be biopsied promptly because these are rarely due to gestational changes [17].

Women experiencing any changes on the palms of their hands, soles of their feet or facial skin can safely undergo an excisional or incisional biopsy, even if they are pregnant [20]. This method provides a definitive histological diagnosis without causing harm to the fetus [46]. Diagnosing melanoma early during pregnancy is key to better outcomes and warrants a low threshold for biopsying suspicious-appearing lesions [47].

## 6. Staging

The importance of accurately staging melanoma cannot be overstated. As stated earlier, pregnancy represents a unique physiologic state, and therefore, it is critical that clinicians utilize the most effective, safest and most practical staging method available when determining a treatment plan and predicting the patient’s prognosis. As such, staging in pregnancy requires clinicians to integrate conventional cancer staging methods with the unique needs of the pregnant patient and fetus [44,46].

Therefore, staging of melanoma during pregnancy includes a complete evaluation of the extent of melanoma, including the use of imaging studies, sentinel lymph node biopsies, etc., to assess the extent of disease progression and metastatic spread [1]. However, as stated above, because pregnancy is a unique physiologic state, the diagnostic processes used to evaluate melanoma must be carefully considered to avoid the potential for causing adverse effects on the developing fetus, particularly radiation exposure [48]. Therefore, when choosing between imaging modalities for staging purposes, clinicians must carefully weigh the advantages of each diagnostic procedure against the potential risks to the fetus, and whenever possible, choose non-ionizing imaging procedures, such as ultrasound or magnetic resonance imaging [39].

### 6.1. Placental Involvement

Placental metastatic spread occurs rarely but is seen in approximately 30% of reported cases of placental metastatic spread. All cases of placental metastatic spread have been associated with concurrent visceral metastatic illness [49]. Therefore, the presence of metastasis in the placenta indicates significant hematogenous dissemination in the mother [49]. To date, only one case of placental involvement has been documented in a large, retrospective French cohort of 22 patients with stage III/IV melanoma who became pregnant [50].

A multicenter retrospective study showed that among the 87 patients with placental or fetal metastases secondary to melanoma, 31% were secondary to melanoma. In addition, 22% of pregnancies complicated by placental metastasis resulted in fetal metastasis. Five of the pregnancies complicated by placental metastasis resulted in the death of the infant. On average, metastatic spread occurred approximately 4.33 months after delivery and primarily involved the central nervous system, liver and lungs.

Therefore, the placenta should be thoroughly examined in all cases of suspected metastatic melanoma during pregnancy to identify any evidence of dissemination [51]. In addition, the newborn should undergo a thorough physical examination, chest radiograph, abdominal ultrasound and evaluation of liver function tests and lactate dehydrogenase levels, particularly if there is evidence of placental involvement, due to the high likelihood of neonatal disease in such cases [52]. Although placental involvement does not necessarily indicate fetal metastasis, it is estimated that between 22 and 25% of infants born with placental metastasis will ultimately succumb to metastatic melanoma [26,51].

### 6.2. Sentinel Lymph Node Biopsy (SLNB) in Pregnancy-Associated Melanoma

The application of sentinel lymph node biopsy (SLNB) as a prognostic tool in the staging of cutaneous melanoma has been established. Research has shown that the use of Technetium-99 m radiocolloids for SLNB mapping is safe during pregnancy. The amount of technetium that the fetus would be exposed to is generally under 5 mGy(miligray), which is well below the commonly accepted threshold for safety. Prenatal radiation exposure is generally considered to be safe at levels under 100 mGy [53,54]. As far as oncology is concerned, the status of the sentinel lymph nodes is a critical prognostic factor for patients with melanomas greater than or equal to 1.0 mm in Breslow thickness. In cases where the melanoma measures 0.8–1.0 mm, with other risk factors such as the presence of ulceration, mitotic rate >1/mm^2^, or the involvement of the vertical growth phase, the decision to perform SLNB may need to be individualized based upon current guideline recommendations [47].

Although SLNB is theoretically safe, the optimal timing of SLNB during pregnancy is still a topic of discussion among clinicians. As SLNB is primarily used as a predictive test rather than as a therapeutic intervention, delay of SLNB until the postpartum period is recommended for many melanomas diagnosed during pregnancy, regardless of the gestational age at diagnosis. Delaying the performance of SLNB does not appear to have a significant impact on the prognosis of these patients. Additionally, if a complete lymphadenectomy is indicated, it will likely require general anesthesia, thus increasing potential risks to both the mother and the fetus [10,52].

It is also very important for clinicians to consider the possible increased exposure of the fetus to additional medications that are used during SLNB. Dyes used to identify the lymphatic pathway in SLNB, specifically isosulfan blue and methylene blue, are classified as category C pregnancy medicines, indicating that there is no information available regarding their teratogenic or reproductive effects. Therefore, these dyes are often avoided during pregnancy [55].

Therefore, the decision to perform SLNB during pregnancy must be made on a case-by-case basis, taking into account the gestational age of the fetus, the characteristics of the tumor, the patient’s desires, and the anticipated effect of the results of the SLNB on the patient’s short-term management. There is a consensus that the use of radiocolloids for SLNB when necessary is safe; therefore, many experts suggest initiating this type of SLNB in the second trimester to minimize the risk to the fetus [56].

### 6.3. Video-Assisted Inguinal Lymphadenectomy (VIL)

Video-Assisted Inguinal Lymphadenectomy has both diagnostic and therapeutic functions, as it allows surgeons to determine if there is involvement of regional lymph nodes and to remove the metastatic nodes, which will guide future treatments and provide better patient outcomes. It is a less invasive approach with fewer difficulties and shorter hospital stays [52].

This alternative to open lymphadenectomy has the potential to lower the morbidity from surgery in pregnant women. It also has the advantage of shortening post-operative recovery time and decreasing potential complications in pregnant women, where these factors are critical. As such, the use of a conservative surgical approach (such as Video-Assisted Inguinal Lymphadenectomy), when combined with proper pre-operative planning and intra-operative anesthetic precautions, can significantly reduce the risk to both mother and fetus [53].

There is documented evidence of successful completion of VIL in a pregnant woman in the 24th week of gestation who had recurrent melanoma. The affected lymph nodes were successfully removed during this procedure, thus demonstrating the feasibility of utilizing advanced surgical techniques during pregnancy when proper precautions are taken during both the anesthetic and surgical phases of care [57].

General anesthesia is most commonly used for Video-Assisted Inguinal Lymphadenectomy. Careful consideration should be given to both the anesthetic agent(s) used and the anesthetic technique utilized to minimize both fetal exposure and maternal complications, especially when treating melanoma in pregnant women. There is documentation of a steep learning curve associated with Video-Assisted Inguinal Lymphadenectomy. Thus, surgeons wishing to utilize this technique must undergo specialized training and have considerable experience with both advanced laparoscopic surgical techniques and the unique physiological changes associated with pregnancy [57]. Video-Assisted Inguinal Lymphadenectomy may be a safe and effective method to diagnose melanoma while minimizing delay in the initiation of aggressive cancer treatment [58].

### 6.4. Imaging Modalities

Imaging plays a crucial role in assessing the extent of melanoma by providing an overview of regional lymph node metastasis and distant metastases that will help guide treatment. However, imaging pregnant women differs from imaging non-pregnant women. When deciding whether to image pregnant patients, physicians have numerous factors to evaluate before imaging, particularly the benefits of the imaging studies versus the risks to the developing fetus. Consequently, the imaging studies selected must meet safe levels of radiation exposure for the fetus and/or use contrast media safely [10,39].

Ultrasound, CT scans and MRI are commonly used in clinical practice for melanoma evaluation. When caring for pregnant patients, the physician must take into consideration the use of ionizing radiation and contrast dyes to the developing fetus and therefore should adjust the imaging strategy for each individual patient [53].

Ultrasound is generally considered the safest initial imaging study. Ultrasound is particularly useful in evaluating superficial lesions and regional lymph nodes. MRI can also serve as a useful tool when evaluating possible metastatic disease during pregnancy, as MRI does not expose the fetus to ionizing radiation. Although MRI can produce excellent anatomical details, most centers do not utilize gadolinium-based contrast agents due to the possibility of the agent crossing the placental barrier and the lack of knowledge about the long-term effect of gadolinium on the fetus [44]. The evaluation of regional lymph nodes continues to play a major role in determining the stage of melanoma. A sentinel lymph node biopsy, when clinically indicated, can provide valuable prognostic information. Due to physiological changes in pregnancy (e.g., increased lymphangiogenesis) that can potentially alter lymph node involvement, this highlights the importance of accurately determining regional involvement in this population [26,59].

In contrast to the above-mentioned imaging studies, PET/CT involves a large amount of radiation to the fetus; thus, it is typically avoided during pregnancy. If a complete body imaging study is required, alternative methods (whole body MRI or PET-MRI) may be employed, as they can provide all necessary staging data while significantly decreasing the amount of radiation exposure to the fetus [53].

## 7. Cancer Treatment in Pregnancy

A team approach to treating melanoma during pregnancy is required, as well as a thoughtful balance between the health of the woman and her unborn child [26]. This involves an assessment of the gestational age, the extent of the melanoma and the potential impact of each type of treatment on both the likelihood of cure for the woman and the development of her fetus [60]. The timing of surgical interventions (especially large local excisions and sentinel lymph node biopsies) typically requires careful consideration; some recommendations support delaying sentinel lymph node biopsy until the postpartum period in selected cases [51]. However, the contemporary literature has demonstrated that sentinel lymph node biopsy utilizing technetium-99 is safe during pregnancy and results in relatively low levels of fetal radiation exposure, far less than the established thresholds of safety [26]. Although there are concerns regarding the exposure of the fetus to radioactive colloid and blue dye used in sentinel lymph node biopsy, this intervention is generally felt to be safe for pregnant women. In fact, a few studies have suggested performing pre-operative lymphoscintigraphy, followed by wide local excision under local anesthesia, and then performing the delayed SLNB post-delivery for women diagnosed with melanoma early in their pregnancy [20,51,61].

Melanoma removal from a pregnant woman is safe and needed for diagnosis. According to the National Comprehensive Cancer Network, tumors measuring 1.01 to 2 mm in diameter should be removed with a 1 to 2 cm margin. This is done using local anesthesia similar to that used for a lymph node biopsy [62].

### 7.1. Surgery

Surgery can be performed at any gestational age when necessary. To lower the risk of having a miscarriage, it is ideal to do interventions in the early second trimester [63]. Surgical treatment can be performed solely under loco-regional anesthesia, as the malignancy is situated within the skin, offering safe and easy surgical access. Lidocaine is preferred since it is FDA category B and does not harm the fetus, even though it crosses the placenta. Moreover, benign tachycardia and vertigo are the primary side effects in pregnant women [47].

A comprehensive analysis of over 12,000 women receiving non-obstetric surgical procedures indicated no increased risk of miscarriage or congenital anomalies, even in cases requiring general anesthesia. Once melanoma has been confirmed, the appropriate investigations are dictated by the tumor stage as per the American Joint Committee on Cancer (AJCC) staging system and the gestational age/trimester [29].

This systematic method makes sure that diagnostic tests and the following treatments are done to lower risks for both the mother and the fetus while also improving cancer outcomes.

### 7.2. Radiation

There are several factors to consider when deciding whether to treat melanoma that occurs during pregnancy with radiation. Generally, single exposures to radiation, such as those resulting from a single-phase CT examination of the abdomen or pelvis, provide less than 50 mGy to the fetus and therefore pose little hazard. The principle of radiological protection, ALARA (As Low As Reasonably Achievable), emphasizes that although there is no “safe” level of ionizing radiation’s stochastic effects, exposure to radiation should always be minimized to the lowest achievable levels. Therefore, it is essential to use imaging studies and therapeutic radiation judiciously during pregnancy [64]. This means that radiation exposure should be kept as low as possible, which shows how important it is to use imaging studies and therapeutic radiation carefully during pregnancy.

The potential hazards to the conceptus from radiation depend on the dose and the gestational age of the embryo/fetus. For example, doses exceeding 100 mGy have been associated with an increased risk of developmental delays in the second trimester; structural malformations during the first trimester; and spontaneous abortion within the first two weeks of pregnancy. While the risks associated with low-dose fetal radiation exposure have been reported to be small, low-dose fetal radiation has also been associated with an increased risk of cancer in both children and adults [65,66].

Radiation therapy is generally feasible in stage IV melanoma if the uterus is placed outside of the radiation field and shielded from scattered radiation. However, this approach will only benefit pregnant women with oligometastatic disease confined to one site, such as a single-site intracranial metastasis. Pregnant patients who require radiation therapy should utilize modern technologies, including proton-beam therapy, intensity-modulated radiation therapy (IMRT), and stereotactic radiotherapy, because they result in minimal fetal radiation exposure while providing adequate therapeutic radiation [66].

### 7.3. Systemic Therapy

The most recent treatment strategies have shifted from targeted therapies to systemic therapy, with immunotherapy being the primary treatment for melanomas that range from Stage IIB through IV, and targeted therapies against BRAF mutations (BRAF-mutated, braf-targeted and mek-targeted). Immunotherapy has been shown to reduce the rate of recurrence and increase the length of time until distant metastasis occurs when administered after surgery [67]. The lack of suitable clinical studies is currently a significant obstacle to assessing whether immunotherapy will be useful in treating PAM. It would certainly provide a means to assess the therapeutic potential of this approach; however, conducting such research on pregnant patients is unethical [68].

Biologically, pregnancy represents a state of maternal immune tolerance to the fetus, which is essentially a semi-allograft. In order to maintain this immunological tolerance, PD-1/PD-L1 and CTLA-4 pathways play an important role. Theoretically, blocking these pathways is risky because it can cause placental dysfunction, placental insufficiency, intrauterine growth restriction (IUGR), preeclampsia or HELLP syndrome, as well as potentially create problems with neonatal autoimmunity. There are extensive reviews in the literature on the topic of using immunotherapy in pregnancy [69].

As previously stated, most ICIs are IgG antibodies (for example, nivolumab and pembrolizumab are both IgG4), which, in theory, can cross the placenta, especially during the second and third trimesters (when IgG levels peak), creating a concern regarding the potential effects of the drug on the fetus/neonate.

Although there is increasing interest in the use of immunotherapy for melanoma, the safety and efficacy of immunotherapy during pregnancy are still largely unknown and pose challenges for widespread use. The use of immunotherapy in pregnant women with advanced melanoma is typically discouraged and often requires multidisciplinary discussion and consideration of terminating the pregnancy in either the first or second trimester to enable additional treatment options [52]. Although there is limited evidence available, several case reports have suggested that certain immunotherapies may be considered for selected patients, primarily when the prognosis for the mother is poor and there are no alternative treatment options [10].

### 7.4. Metastases and Recurrence of Melanoma

Malignant melanoma needs appropriate treatment modalities due to its potential to induce satellite metastases, distant metastases, and unique transit. The primary lesion and any satellite metastases, which are those that are in the skin around it, should be excised [70]. Even though they can be surgically removed and are easy to get to, transit metastases to sentinel lymph nodes (SLNs) are more difficult because the patient has to be put under general anesthesia. Adaptive changes in the mother’s physiology during pregnancy, such as tachycardia, hypercoagulability, hypotension, a higher risk of bleeding, and less action in the lower esophageal sphincter (LES), may make it harder to give her more anesthesia [27]. Melanoma may be unresectable if the tumors are in places that are hard to reach or if the cancer has spread to other parts of the body. This means that the tumors cannot be completely eliminated during surgery. In some circumstances, further treatments like immunotherapy and radiotherapy may be needed. Unfortunately, unlike melanoma outside of pregnancy, treatment options may be fewer [71,72].

### 7.5. Consequences for Both Mother and Child

Most research assessing the impact of melanoma treatment on fetuses was performed on animals. Because of this, we do not know how many of them affect human pregnancies, and some of the results are not clinically important. There are many problems that can happen when you use anesthetics, antibiotics, imaging, or invasive treatments [73].

Notably, 8.7% of the groups being studied had preterm labor, and 5.1% had low birth weight. In comparison, 10% and 8% of the overall population were impacted. A birth weight below 2500 g and gestational age exceeding 37 weeks were associated with a 0.9 risk ratio for melanoma, whereas a reduced gestational age corresponded to a 0.8 risk ratio. In conclusion, the incidence of melanoma identified during pregnancy did not connect with any of the three specified criteria [74].

There is little evidence to conclude that a poorer prognosis is associated with melanoma diagnosed during pregnancy. Some research, however, reveals a 17% rise in obstetric patient mortality. To determine the danger, further work is necessary [52]. Because the prognosis of PAM is uncertain, careful counseling is needed to address the risk of recurrence and the importance of individualized oncologic follow-up. Shared decision-making is essential to balance each patient’s reproductive goals with the safety of the oncologic care plan [25].

## 8. Discussion

Melanoma and pregnancy both undergo significant physiological alterations; therefore, it is vital to provide a detailed explanation of the previous literature, assess gaps in current knowledge, and outline critical areas of study. Some studies have reported higher mortality rates among pregnant women with melanoma compared to non-pregnant women; however, there are many methodological issues and variable definitions of what constitutes “pregnancy-associated” melanoma that limit the comparison of mortality rates [17,75]. Definitions of pregnancy-associated melanoma vary among studies and make comparisons of mortality rates problematic [39]. Furthermore, studies are mostly retrospective and do not adequately control for confounding variables, such as tumor thickness and stage of melanoma at the time of diagnosis, which significantly affect prognosis regardless of whether the woman was pregnant [44,51]. While some meta-analyses report similar mortality rates for pregnant and non-pregnant women with melanoma, other meta-analyses report a greater likelihood of mortality for pregnant women than for non-pregnant women, after controlling for age and stage of disease [10,25]. These discrepancies highlight the need for a uniform definition of “pregnancy-associated melanoma” and a prospective study to conclusively define the potential prognostic effects of pregnancy on melanoma [25,26]. Tumor characteristics are a component of discussions related to the effect of pregnancy on melanoma prognosis. Some studies have shown varying levels of tumor thickness and extent of lymph node involvement among pregnant women, while others show no significant differences in primary tumor depth and/or aggressiveness [3,31]. Most initial studies demonstrating a poor prognosis for pregnant patients with melanoma were without adequate controls and did not take into consideration other important prognostic markers, such as tumor depth, and therefore may have produced biased results [47].

The controversial potential of hormone-related aspects of pregnancy on melanoma development continues to be discussed. A key source of debate in the field of pregnancy-associated melanoma (PAM) is the disconnect between the biological plausibility of hormone-mediated pathways and the clinical evidence demonstrating these pathways are involved in melanoma development. While many laboratory-based studies suggest that estrogen and/or progesterone can induce melanoma cell growth and viability in certain conditions, these studies were conducted using in vitro systems, animal models, or other non-clinical systems, which do not represent how hormones function in humans. Therefore, the clinical evidence does not support that exposure to endogenously produced hormones during pregnancy impacts the prognosis of patients with melanoma. It appears that while there are biologically plausible ways that hormones could interact with melanoma cells during pregnancy, the clinical significance of such interactions has not been clearly defined [4,6,26]. Therefore, it would be more appropriate to view mechanistic data as possible explanations for observed phenomena, rather than as definitive evidence of a distinct “pregnancy-driven” melanoma phenotype.

Collectively, the evidence supports a hierarchical understanding of the relationship between pregnancy and melanoma: preclinical studies suggest that there are biologically plausible ways in which pregnancy-related physiological changes may interact with melanoma; clinical studies demonstrate conflicting results regarding the relationship between pregnancy and melanoma; and contemporary reviews often conclude that pregnancy is not a proven independent adverse prognostic factor for melanoma, especially when melanoma is localized. By framing the literature in a hierarchical fashion, we avoid over-interpreting the evidence and better reflect our current understanding of the topic.

From a purely immunologic standpoint, pregnancy involves a condition of relatively immune tolerance, which is mediated through various immune checkpoint pathways, including CTLA-4 and PD-1/PD-L1. Although these mechanisms raise theoretical issues about enhanced tumor immune evasion, at present, there is no clinical evidence to suggest that the immunomodulation associated with pregnancy will result in enhanced melanoma aggressive behavior [27]. These mechanisms, however, may be relevant when evaluating systemic therapy options in the management of advanced disease.

Melanomas associated with pregnancy are particularly difficult to manage due to the need to balance maternal health with fetal health. A multidisciplinary approach is the best way to care for a patient with pregnancy-related melanoma, which is difficult to diagnose, stage, and treat [76]. Therefore, using a tailored, multidisciplinary approach will allow for a better understanding of the mother’s needs and perspective [77].

Excisional biopsies with lidocaine to confirm a suspicion of melanoma can be safely performed early in pregnancy, regardless of gestational age. By adopting this approach, PAM’s prognosis can be improved by minimizing delays in diagnosis and treatment [78].

Pregnant women should regularly examine their skin for any changes, such as new moles or changes in the size, shape, or color of moles they already have. They should inform their physician if they observe any changes that concern them [70]. Early detection and appropriate management of melanoma improve survival [79]. One of the main fears is that because of physical changes in the skin that occur during pregnancy, PAM may not be diagnosed promptly enough. In addition, a complete risk/benefit evaluation must be conducted prior to the selection of diagnostic procedures, since pregnancy imposes limitations upon imaging and histologic assessments [61]. The optimal time to perform a sentinel lymph node biopsy (SLNB) during pregnancy remains controversial; however, SLNB is an important staging procedure, particularly for tumors with a Breslow thickness of 1 mm or more. Despite the fact that technetium-99 m (99mTc) radiotracers are considered safe, concerns regarding exposure to the developing fetus may result in postpartum delays in staging unless the staging is urgently needed [53]. However, video-assisted sentinel lymph node biopsy represents an alternative and potentially less risky means of staging, which would facilitate timely staging for optimized therapy, but it is site-dependent and not a standard of care [80]. As our understanding of the molecular nature of melanoma grows, including its hormonal modulation during pregnancy, new targeted therapies and individualized treatment strategies will evolve [10].

The primary form of treatment for localized PAM, surgical excision, can be safely performed as a wide local excision at any point during the pregnancy, whereas advanced or metastatic melanoma does present significant problems for the pregnant patient [26]. The risk for systemic therapy (i.e., traditional treatments such as immunotherapies and targeted therapies) for placental insufficiency, restricted fetal growth, and/or an immune response by the fetus that could lead to neonatal complications is increased with the administration of these therapies during the first and second trimester. As a result of this, treatment decisions for patients diagnosed with advanced PAM will involve a multidisciplinary, individualized assessment in a specialized center and will include careful consideration of the gestational age, the burden of the disease, and the prognosis of the mother. Often, systemic therapy is delayed until after delivery when possible [71,72].

Much is being learned about pregnancy-associated melanoma (PAM), but there continue to be some significant areas where there is a lack of information. The majority of what we have been able to learn has come from retrospective studies that were limited by smaller numbers of subjects; therefore, they may have introduced selection bias and made it difficult to determine if pregnancy was directly related to melanoma outcome. Additionally, little is known regarding how the changes in hormones and the immune system associated with pregnancy can affect the progression of melanoma. Multicenter prospective studies and larger population-based analyses will need to be conducted in the future to provide a clearer understanding of the mechanisms involved in PAM and improve evidence-based treatment recommendations for pregnant women diagnosed with melanoma.

## 9. Conclusions

Pregnancy-associated melanoma can be difficult to manage due to the need to weigh the best maternal oncology treatment against fetal risk. Currently, there is no clear evidence that pregnancy independently worsens the prognosis of melanoma. While various physiological alterations present in pregnant women (hormonal, immunologic, and vascular) provide a biological rationale for potential alterations in tumor behavior during pregnancy, the clinical relevance of these alterations has yet to be proven. The apparent poor outcomes reported in several studies are more likely due to delays in diagnosing the melanocytic lesion, detection after delivery, and/or differences in presenting stages and study methodology. Therefore, suspicious melanocytic lesions identified in pregnancy should be evaluated immediately, and management should focus on early diagnosis, utilize oncologic principles applied to pregnancy, and involve multidisciplinary decisions.

## Data Availability

The data presented in this study are available on request from the corresponding author.

## Abbreviations

The following abbreviations are used in this manuscript:

PMA	Pregnancy-associated melanoma
SLNB	Sentinel lymph node biopsy
CMM	Cutaneous malignant melanoma
